# Fluoride transport in *Arabidopsis thaliana* plants is impaired in Fluoride EXporter (FEX*)* mutants

**DOI:** 10.1007/s11103-023-01413-w

**Published:** 2024-02-11

**Authors:** S. Lori Tausta, Kathryn Fontaine, Ansel T. Hillmer, Scott A. Strobel

**Affiliations:** 1 Department of Molecular Biophysics and Biochemistry, Yale University, New Haven, CT 06510 USA; 2 Institute of Biomolecular Design and Discovery, Yale University West Campus, West Haven, CT 06516 USA; 3 Department of Radiology and Biomedical Imaging, Yale School of Medicine, 330 Cedar Street, New Haven, CT 06519 USA; 4 Yale PET Center, Yale University, 801 Howard Avenue, New Haven, CT 06510 USA; 5 Department of Biomedical Engineering, Yale University, 17 Hillhouse Avenue, New Haven, CT 06511 USA; 6 Department of Psychiatry, Yale School of Medicine, 300 George Street, New Haven, CT 06510 USA

**Keywords:** Fluoride, Tolerance, Positron emission tomography, Toxicity

## Abstract

Fluoride is an environmental toxin prevalent in water, soil, and air. A fluoride transporter called Fluoride EXporter (FEX) has been discovered across all domains of life, including bacteria, single cell eukaryotes, and all plants, that is required for fluoride tolerance. How FEX functions to protect multicellular plants is unknown. In order to distinguish between different models, the dynamic movement of fluoride in wildtype (WT) and *fex* mutant plants was monitored using [^18^F]fluoride with positron emission tomography. Significant differences were observed in the washout behavior following initial fluoride uptake between plants with and without a functioning FEX. [^18^F]Fluoride traveled quickly up the floral stem and into terminal tissues in WT plants. In contrast, the fluoride did not move out of the lower regions of the stem in mutant plants resulting in clearance rates near zero. The roots were not the primary locus of FEX action, nor did FEX direct fluoride to a specific tissue. Fluoride efflux by WT plants was saturated at high fluoride concentrations resulting in a pattern like the *fex* mutant. The kinetics of fluoride movement suggested that FEX mediates a fluoride transport mechanism throughout the plant where each individual cell benefits from FEX expression.

**Supplementary Information:**

The online version contains supplementary material available at 10.1007/s11103-023-01413-w.

## Key Message

Using positron emission tomography (PET) to monitor the movement of F^−^ in wild type and *Fluoride EXporter *mutants revealed the inability of the mutant to keep F^−^ out of cells.

**Supplementary Information:**

The online version contains supplementary material available at 10.1007/s11103-023-01413-w.

## Introduction

Fluoride (F^−^) is abundant in the environment, but is toxic to plant and animal life. Fluoride is naturally released into the biosphere by weathering of fluoride-containing minerals and from volcanoes and marine aerosols (Symonds et al. 1988; Weinstein and Davison 2004). Fluoride is released by coal burning and manufacturing processes involving metal smelting or chemical reduction of fluoride-containing minerals. Fertilizers used to enhance growth conditions for crops contain fluoride, initially from fluorapatite-rich phosphate rock, which results in fluoride accumulation in the soil (Anbuvel et al. 2014; Ramteke et al. 2018). As a result, plants are exposed to fluoride through the air, water, and soil. Different plant species vary in their fluoride uptake, accumulation, and tolerance. Some plants, such as *Camilla sinensis* (tea), can tolerate high levels of fluoride without ill effect, but most plants are sensitive at concentrations of less than 20 µg F/g dry weight (Jacobson et al. 1966). Other than a few species that produce toxic organofluorides, fluoride is not required biologically and is harmful to plants.

Fluoride from the soil enters the root apoplast mainly by passive diffusion (Garrec and Letourneur 1981; Mackowiak et al. 2003). Cell walls are the first barrier to cell entry, in part because the Ca^2+^ found there complexes with the negative fluoride ion and forms insoluble CaF_2_ (Miller et al. 1986; Ruan et al. 2004; Cai et al. 2014). In addition to the cell wall, the negatively charged cell membrane repels fluoride ions. However, the cell membrane is susceptible to HF, the conjugate acid of fluoride, prevalent at lower pH (pK_a_ = 3.4). To enter the transpiration stream, fluoride must either travel through the symplast to the vasculature or pass through the root endodermal barrier. However, the root endodermis does have discontinuities through which fluoride can enter the transpiration stream (Davison et al. 1985; Takmaz-Nisancioglu and Davison 1988). The fluoride concentration found in the leaves directly reflects the concentration of fluoride in the growth medium and the amount of water flow through the plant, consistent with the ability of fluoride to by-pass the endodermis (Takmaz-Nisancioglu and Davison 1988; Banarjee and Roychoudhury, 2019). Once in the xylem, fluoride has been shown to move with the transpiration stream and accumulate at the tips of leaves (Elloumi et al. 2005; Hong et al. 2016).

Fluoride toxicity is manifest in a variety of ways, including complexation with cations like Ca^2+^ and Mg^2+^, cell wall disintegration, enzyme inactivation, and photosynthesis inhibition (Weinstein and Davison 2004; Banariee and Roychoudhury, 2019). Fluoride can also directly react with and damage proteins and cell membranes, interfere with phosphorylation, and depolarize membranes (Hong et al. 2016; Sharma and Kaur 2018; Gadi et al. 2021). Detrimental levels ultimately cause chlorosis, leaf burn, and tissue necrosis. Every stage of plant growth and tissue can be harmed by fluoride (Hong et al. 2016).

Despite being prevalent and toxic, the biochemical basis of fluoride resistance was only recently discovered. A fluoride transporter, originally found in bacteria (fluc- FLUoride Channel) and yeast (FEX, Fluoride EXporter), is an important mechanism of fluoride tolerance in plants (Li et al. 2013; Stockbridge et al. 2013; Berbasova et al. 2017). Both fluc and FEX specifically and rapidly efflux fluoride ions to keep the concentration within cells at nontoxic levels. For example, a yeast strain lacking FEX is 1000-fold more sensitive to fluoride in the growth media resulting in fluoride sensitivity at concentrations commonly found in municipal drinking water (60 μM; Li et al. 2013). Fluc is at least 1000-fold selective for F^−^ over Cl^−^, the next closest halide (Stockbridge et al. 2013) and FEX from yeast or *Arabidopsis* is also highly selective (Tausta et al. 2021).

FEX homologs are found in all plants for which sequence information is available. Plant FEX homologs from different species were able to rescue a yeast FEX mutant grown in fluoride. This established that the putative FEX proteins from plants that are as diverse as moss and angiosperms are active fluoride transporters (Berbasova et al. 2017; Song et al. 2020; Tausta et al. 2021).

There is one *FEX* gene in *Arabidopsis*, which is expressed at a low level in most tissues ((BAR-Arabidopsis eFP browser; Schmid et al. 2005; Winter et al. 2007; Tausta et al. 2021). *Arabidopsis* with a *fex* knock-out mutation cannot tolerate even small amounts (~ 1 µM) of fluoride in the growth substrate resulting in yellowing leaves, stunted growth, and infertile flowers (Tausta et al. 2021). Higher concentrations of fluoride lead to necrosis and death of the plants. When grown in the presence of fluoride, the flowers at the apex of the mutant plant were shown to accumulate higher concentrations of fluoride when compared to wild type (WT) FEX flowers (Tausta et al. 2021). Conversely, overexpression of FEX resulted in fluoride tolerance at elevated concentrations (Zhu et al. 2019; Song et al. 2020; Tausta et al. 2021). Thus, FEX is necessary and sufficient for fluoride resistance in *Arabidopsis*.

This raises the question of how FEX functions to protect plants from fluoride toxicity. A few different possibilities can be imagined. One model is that fluoride ions could be detoxified by localization to the vacuole, a mechanism called ‘vacuole detoxification’, for which there is some evidence in the hyperaccumulator tea (*Camilla sinensis*; Gao et al. 2014). Many toxins and excess ions are shunted to the plant vacuole where they can be stored or detoxified. For instance, Cl^−^ is localized into barley vacuoles (Martinoa et al. 1986) as are heavy metals such as cadmium and zinc (Sharma et al. 2016; Clemens and Ma 2016). A second model is that FEX functions in the plant roots to exclude fluoride from the transpiration stream, a strategy termed ‘avoidance’. It is a known strategy for metal tolerance (Tognacchini et al. 2020). A third possibility involves FEX expression in specific cell types in such a way that it directs fluoride to expendable tissues, such as the plant leaves, a model termed ‘selective tissue targeting’. For example, zinc accumulates in the leaf trichomes of *Arabidopsis thaliana and A. halleri* (Zhao et al. 2000; Ricachenevsky et al. 2021). A fourth model is that each cell utilizes FEX to efflux accumulated fluoride out of its own cytoplasm, thus continually moving fluoride into and through the transpiration stream in the plant. Fluoride could then be exuded through the hydathodes and/or released at the stomata with water. This model is effectively ‘each cell for itself’ and is most similar to what happens in single-celled systems like yeast (Li et al. 2013; Stockbridge et al. 2013). These four mechanisms are not necessarily exclusive of each other and other variations can be imagined. The FEX knockout mutation in *Arabidopsis* provides an opportunity to distinguish between these models and to explore how the fluoride transporter protects a multicellular organism from fluoride toxicity.

Fluoride has a short-lived isotope, [^18^F]fluoride, that is a positron emitter with a half-life of 110 min. This makes it possible to use positron emission tomography (PET) imaging to monitor fluoride kinetics within a plant in real time during the first few hours of fluoride exposure. PET was first employed for the study of fluoride movement in soybean using planar coincidence detectors of [^18^F]fluoride (McKay, 1988). Subsequent work with a more advanced positron-emitting tracer imaging system (PETIS) advanced these findings to show that [^18^F]fluoride transport in soybean was faster than [^15^O]H_2_O, and, thus, may not be equivalent to water transport (Nakanishi et al. 2001). Currently, PET scanners have developed sufficient sensitivity to provide three-dimensional measurements of radiolabeled tracers in the thinner tissues of plants (Converse et al. 2015; Fatangare and Svatos, 2016; Schmidt et al. 2020; Mincke et al. 2021). Specifically, monitoring [^18^F]fluoride via PET has confirmed the ability of plants to transport fluoride in the transpiration stream (McKay et al. 1988; Hubeau and Steppe 2015).

We set out to determine how FEX affects fluoride uptake, movement, and accumulation by analyzing wildtype (WT) and mutant FEX (*fex*) *Arabidopsis* plants using PET. We did this under a variety of experimental conditions to understand the role FEX plays in protecting the plant from fluoride toxicity. These observations provide experimental evidence to distinguish between several roles for FEX in fluoride resistance. The results reported here support the ‘each cell for itself’ model of fluoride movement within the plant. We found that the presence of FEX did not greatly affect initial fluoride uptake into the plant, but fluoride movement in the transpiration stream was severely inhibited in the FEX mutant.

## Materials and methods

### Plant material and treatments

*Arabidopsis thaliana* plants were grown to flowering in growth media (2ProMix Micorrhizae:1peat moss) or hydroponically in 1/4X Hoagland’s solution (Sigma–Aldrich) in chambers with 12 h light, 60% relative humidity. In this study the *A. thaliana FEX* mutant, represented here as *fex*, was a frameshift mutation described previously and the wildtype, WT, was the Columbia ecotype of the *A. thaliana* parent used to create the frameshift (Tausta et al. 2021). The mutant rescue was obtained by transforming the heterozygous mutant (*FEX/fex*) with pADH1:YFP-AtFEX which was made using Gateway to move the AtFEX cDNA cloned in pENTR/d into pADH1:YFP-GW (Michniewicz et al. 2015). T2 and T3 lines were genotyped for both presence of the transgene and the parental *fex* mutation using dCAPS (Tausta et al. 2021). For most PET experiments, pairs of flowering plants, WT and *fex*, were chosen to be as close in development as possible. Occasionally tissues were trimmed to create clear boundaries between the two adjacent plants on the scanning surface. It was found that trimming should be done 24 h ahead of the scan to prevent [^18^F]fluoride from leaking from the wound. Transpiration rates were measured with a Leaf Porometer Model SC-1(Decagon Devices). Treatments of either NaF or NPPB (5-nitro-2-(3-phenylpropylamino) benzoic acid; Sigma–Aldrich) were added to the growth media 24 h before scanning and diluted in 0.01% MES buffer during PET.

### Confocal microscopy

Laser Scanning Confocal images of YFP and chlorophyll autofluorescence were acquired sequentially with an excitation at 488 nm and signal detection 505–550 nm and 650–700 nm respectively on a Leica SP8. All instrument settings remained the same between frames.

### [^18^F]Fluoride production

[^18^F]Fluoride was produced with a GE PETTrace cyclotron (GE Medical Systems, Uppsala, Sweden) by bombardment of 16.5 MeV protons onto a target of pure ^18^O-enriched water to trigger ^18^O(p,n)^18^F reactions. Irradiated target water with [^18^F]fluoride was diluted to a target concentration of 37 MBq (1 mCi, ~ 1.5 nM) in 0.5 mL of 0.01% MES buffer pH 5.4–5.6 for subsequent use in plant imaging procedures.

### PET/CT

**Fig. 1:**
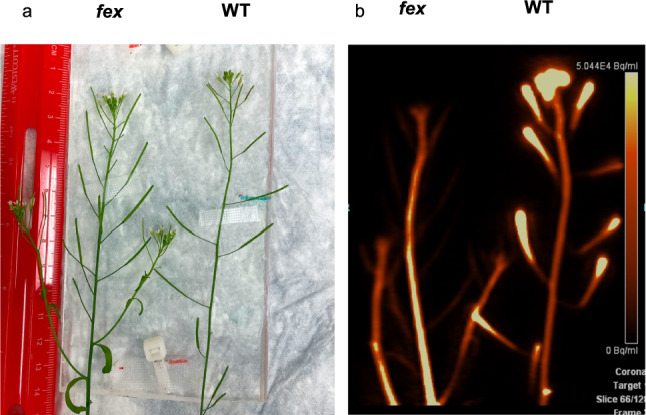
An example of plant set-up and resulting PET scan. **a** Plants are secured to an acrylic plate as shown here and then covered with gauze. Glare from plastic has been removed from this picture. **b** The resulting PET image from all frames. The color bar indicates activity concentration ranging from 0–50.4 kBq/mL. Here, the WT plant has accumulated [^18^F]fluoride in all terminal tissues while in the *fex* plant the [^18^F]fluoride is at the base of the stem after 3 h

Experiments were performed using an Inveon small animal PET/CT system (Siemens Medical Solutions). Trials were run at times corresponding to 5–8 h of the light cycle of plants grown in long day chambers. Plants were secured on an acrylic sheet with gauze to provide water-equivalent material to interact with positrons, increasing coincident events for detection while also allowing for adequate air flow across the top of the plant. The plate was placed horizontally atop a shuttle bed pallet. Roots or stems were left hanging off the end in order to facilitate the administration of [^18^F]fluoride, allow for buffer exchange, and to keep the tube with [^18^F]fluoride out of the scanner field of view (Fig. 1). During acquisition of computed tomography (CT) images for positioning and attenuation correction, plant roots or stem ends were left submerged in 0.01% MES buffer. Immediately following, the PET emission acquisition began with the administration of the [^18^F]fluoride solution in 0.01% MES buffer to the roots or freshly cut floral stem. After 10 min of [^18^F]fluoride uptake, the [^18^F]fluoride solution was removed, roots or stem were either quickly patted dry with gauze or the stem cut, and placed in a fresh tube with 0.01% MES buffer. An LED photolight (Neewer) was used to illuminate the Inveon cavity to 55 µmol/m^2^s. Emission data were acquired for up to 3 h under laboratory conditions of 23–25 °C and 35–50% RH. The radioactivity in the plant, buffer solution, and any containers that had contact with radioactivity were measured using a Capintec CRC-15r dose calibrator after each imaging session.

List mode emission data were binned into discrete time frames (10 × 90 s; 9 × 300 s; 600 s for remainder) and reconstructed with MAP OSEM using two iterations and 25 subsets, including corrections for random events, attenuation, and scatter to yield images with voxel sizes of 0.776 × 0.776x0.796 mm. Reconstructed images were decay-corrected to time of [^18^F]fluoride administration. Regions of Interest (ROIs) were defined on images summed over all timepoints by calculating the gaussian distribution of activity concentrations in profile perpendicular to the plant stem and constraining to voxels within the full-width at two thirds maximum of the perpendicular profile. The ROI position was labeled by the midpoint in distance from the bottom of the field of view using the Inveon Research Workplace software (Siemens). Time activity curves (TACs) were extracted and analyzed both with raw activity concentration values and by normalization to the highest value using Prism 9. Raw PET scans are available upon request. The number of scans for different experimental conditions is documented in Supplemental Table 1.

Kinetic modeling of [^18^F]fluoride dynamics was best achieved with a finite bolus pulse of fluoride moving through the plant. A sharper initial bolus provided better kinetic properties for quantification. Previous work achieved this by administering drops of tracer to the cut petiole of plants (Converse et al. 2015), however, this method was not possible in *Arabidopsis* due to the technical limitation of the plant’s small diameter ($\le$ 1 mm). Thus, to balance sufficient activity for detection with a finite bolus pulse, the base of the plant was placed in [^18^F]fluoride solution for 10 min. Several methods were tested to achieve a fluoride pulse. This included: (1) absorption through the roots followed by washing in buffer, (2) absorption through the roots followed by cutting off roots and rosette leaves before placing the stem in fresh buffer, (3) absorption through a cut stem followed by recutting the stem and washing in buffer. The final method provided the sharpest bolus pulse of [^18^F]fluoride and was used for quantitative analyses.

For analysis of dynamic [^18^F]fluoride data, two primary outcomes were estimated. First, the rate of initial transport up the stem was calculated by dividing the distance of the first evident trace of radioactivity by time. Second, the rate of [^18^F]fluoride clearance from the stem was estimated by fitting the dynamic data from 30 min post-‘injection’ to the end of the scan with a sum of either one or two decaying exponential functions and constant background (optimal model determined with F-test) and taking the slowest exponential decay rate. The resulting curve fits for some of the WT cut stem scans are shown in Sup. Figure 1a as an example. The resulting value described the terminal rate of radioactivity clearance (negative values) in the region of interest (ROI; units min^−1^). Statistical analysis consisted of two-tailed unpaired t tests and Dunnett’s one-way ANOVA using Prism 9.

## Results

### [^18^F]fluoride administration and imaging in ***Arabidopsis*** floral stems

To understand the role that FEX plays in protecting the plant from fluoride toxicity, PET was used to determine if [^18^F]fluoride localization and dynamics were different between WT and FEX mutant (*fex*) *Arabidopsis* plants. Previously, the *fex* mutant was found to be much more susceptible to fluoride in the growth media becoming stunted, exhibiting tip burn, and unable to produce viable seeds (Tausta et al. 2021). However, *fex* plants could be grown without any fluoride-induced phenotypes in hydroponics where no fluoride was present. Growth in hydroponics allowed the comparison of WT and mutant plants at the same age with no previous exposure to fluoride.

*Arabidopsis* presented significant challenges to PET imaging studies due to the small diameter of its stems (Schmidt et al. 2020). We first tested the feasibility of detecting [^18^F]fluoride in the *Arabidopsis* stem tissues and the ability of [^18^F]fluoride to enter the plant via the roots. Following an initial CT scan used for positioning and feature identification, the plant roots were placed in a tube containing [^18^F]fluoride in buffer just out of the field of view. After 10 min, the roots were returned to a tube with plain buffer and the floral stems were monitored. There was ample detected signal in the vegetative tissues (Fig. 1). Movement of the [^18^F]fluoride bolus from the roots to the aerial parts of the plant was evident. The source buffer showed negligible loss of radioactivity after administration. These results were consistent with observations made with larger plants which established that movement of [^18^F]fluoride bolus within plant tissues could be monitored by PET (*Glycine max*, McKay et al. 1988; rice, Kang et al. 2009; *Brassica oleracea*, Converse et al. 2015; tea, Niu et al. 2020).

The patterns of fluoride movement were distinctly different between the WT and *fex* plants near the base of the stem. In WT plants with roots intact, there was a sharp increase in [^18^F]fluoride at the bottom of the floral stem (WT15: 15 mm from the bottom of the field of view) that continued throughout the 10 min pulse (Sup. Figure 1b). This was followed by a gradual decrease in [^18^F]fluoride consistent with washout of [^18^F]fluoride from the stem section. In contrast, the initial appearance of [^18^F]fluoride in the equivalent floral stem section of the mutant (*fex*15) was slightly slower. Upon reaching its peak, it did not appear that any of the accumulated [^18^F]fluoride cleared out of the tissue segment at later time points during the 180 min of the experiment (Sup. Figure 1b).

Root application of the bolus confirmed the ability of [^18^F]fluoride to enter the transpiration stream through the root tissue bypassing the endodermis. This was consistent with other studies where [^18^F]fluoride supplied to the roots of tea (Niu et al. 2020) and cowpea (Furukawa et al. 2001) that also showed upward mobility into the stem and leaves. The observation that the fluoride entered both WT and *fex* with equally efficiency (WT: 2.9 $\pm$ 1.4 (μCi/g)/μCi bolus, *fex*: 2.2 $\pm$ 0.9 (μCi/g)/μCi bolus), suggested that the main role of FEX was not to keep fluoride out of the root tissue. Thus, while FEX is necessary for fluoride tolerance, it does not act primarily by preventing fluoride from entry via the roots under these conditions.

**Fig. 2:**
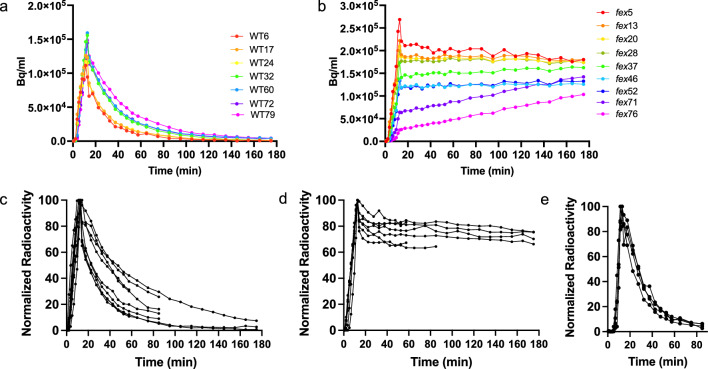
Time activity curves (TACs) resulting from floral stem application of [^18^F]fluoride. **a** All ROIs from the floral stem of a single WT plant which received activity at the bottom of the stem. Labels denote the genotype and the height in mm at the middle of the ROI as measured from the bottom of the field of view. **b** All ROIs from the floral stem of *fex* plants which received activity at the bottom of the stem. **c** All results from PET scans with WT stem application. The TACs shown are from the ROI closest to 20 mm and normalized to the time with the highest radioactivity. **d** All *fex* stem application TACs represented by the ROI closest to 20 mm and normalized to the time with the highest activity. **e** ROIs from four replicates of pADH1:YFP-AtFEX rescued *fex* plants normalized as in **b** and **d**

Application of [^18^F]fluoride to roots followed by washing resulted in continued accumulation of [^18^F]fluoride in both plant genotypes throughout the scanning period (1.5 h). This implicated continual uptake of [^18^F]fluoride from residual radioactivity retained in or on the roots. To better observe the differences in [^18^F]fluoride movement between the WT and mutant plants without the continuous introduction of radioactivity from the roots, which we established was not the main mechanism for resistance, two other methods were tested. First, the [^18^F]fluoride bolus was applied to the roots, followed by cutting off the roots and rosette leaves and placing the stem in cold buffer. Application of the bolus to the roots followed by trimming resulted in a sharper bolus pulse and washout (Sup. Figure 1c). The resulting time activity curve (TAC) from cutting off the roots allowed evaluation of the root contribution, but resulted in a cleaner washout without further contribution of [^18^F]fluoride from the roots after the pulse. The second procedure bypassed the roots by adding the bolus to the cut stem and then recutting the stem and placing it into cold buffer. This resulted in further sharpening of the TAC patterns without altering [^18^F]fluoride dynamics (Fig. 2). This result also confirmed that removal of the roots did not change the pattern of movement or accumulation in the vegetative tissues of either the WT or *fex* plants. Application of the bolus to a cut stem or petiole has been used previously in rice (Kang et al. 2009), Brassica (Converse et al. 2015), and soybean (McKay et al. 1988; Kume et al. 1997). One of these two methods was used for further experiments and kinetic modeling depending on whether the contribution of the root tissue was being tested or not. Removing the roots either before or after the [^18^F]fluoride bolus resulted in patterns of [^18^F]fluoride movement consistent with those seen when the roots were left intact, but were more readily quantified.

### WT and *fex* stems have different fluoride dynamics

There were distinct differences between [^18^F]fluoride movement in the stems of WT and *fex* plants. Within the WT stem, the bolus of [^18^F]fluoride entered during the 10 min pulse and then progressed quickly to higher sections of the plant. Even at the highest region of the floral stem, the amount of [^18^F]fluoride peaked after 12 min and then decreased by 20 min (Fig. 2a, Sup. Fig. S2). For example, in Fig. 2A the stem region of interest (ROI) at 24 mm (distance from the bottom edge of the field of view) in the WT plant (WT24) peaked around 12 min and only 1% of the maximum radioactivity remained at 90 min. In contrast, within the *fex* floral stem, the majority of the radioactivity remained in the lower stem section and did not continue to travel up the stem (Fig. 2b). In the case of the *fex* stem at 20 mm (*fex*20) 83% of the highest [^18^F]fluoride peak count was still present at 90 min (Fig. 2b). In fact, the lowest *fex* stem section retained the highest amount of radioactivity. This is in contrast to the WT where the lowest stem section had the least amount of radioactivity after 90 min. This is evident in the video of the time course (Sup. Movie1) of the scan shown in Fig. 1 and in the resulting time activity curves (Fig. 2). At the latest time frames, the upper sections of the *fex* stem (*fex*71 and *fex*76) still showed a slow increase in [^18^F]fluoride. This is also in contrast to the WT, which showed a decrease in radioactivity in these regions at later time frames, similar to the bottom of the stem. Note that rainbow colored curves, which denote progressively higher sections of the stem, are reversed between the WT and *fex* plants (Fig. 2). Biological replicates confirmed these different patterns across multiple test plants (Fig. 2c and d).

The mutant phenotypes of the *fex* plants were fully rescued with a YFP tagged *AtFEX* construct under the control of the *ALCOHOL DEHYDROGENASE1* (*ADH1*) promoter (Michniewicz et al. 2015). Under our growth conditions of very low fluoride, robust YFP fluorescence as detected by confocal microscopy was seen in most vegetative plant cells (Sup. Fig. S3), and less fluorescence in root cells. This pattern of expression was sufficient to rescue mutant plants such that they were able to produce viable seeds. When rescued plants were given [^18^F]fluoride, the time activity curves were indistinguishable from that of the WT, confirming that rescue was reliant on FEX expression (Fig. 2e).

It was possible that a build-up or malposition of fluoride ions due to loss of FEX caused a decrease in transpiration in the mutant which would appear as a lack of fluoride movement. Although the bolus of [^18^F]fluoride was only ~ 1.5 nM, this concentration might be enough to inhibit stomatal opening. However, when both genotypes were grown in media with a low amount of fluoride and the mutant showed the phenotypes of fluoride sensitivity, the average transpiration rate was not different from WT (Sup. Fig. S4).

### [^18^F]Fluoride kinetic properties in WT and ***fex*** stems

**Fig. 3:**
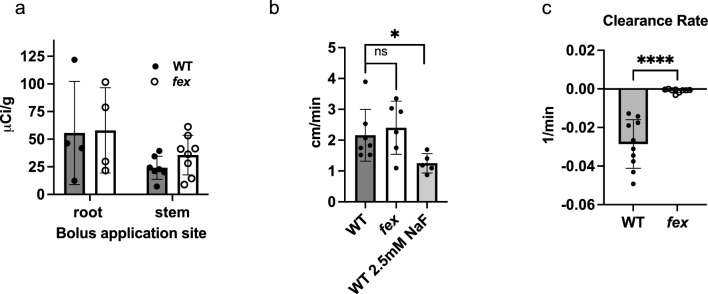
Determination of the amount and speed of [^18^F]fluoride movement in floral stems of WT and *fex* plants. **a** The amount of radioactivity found in the stem tissue after the scan when the bolus was applied to either the roots or the stem. The *fex* stems had comparable radioactivity to the WT stems. **b** The initial velocity of the [^18^F]fluoride up the stem in WT versus mutant was similar, but the addition of 2.5 mM NaF to WT plants slowed the movement. **c** Clearance rate (min^−1^) values of stem [^18^F]fluoride application ROIs around 20 mm in WT and mutants. Here, ns is not significant, * is P < 0.05, **** is P < 0.0001 and are the results of the unpaired two-tailed t test

Application of [^18^F]fluoride to the cut stem provided a suitable bolus to evaluate kinetic properties for fluoride uptake. This method was used for the quantification of [^18^F]fluoride initial velocity and clearance. The entry of [^18^F]fluoride into the vasculature of the cut stem was fast with little lag across all plants whether WT or *fex*. The total amount of radioactivity measured in the stems of either WT or *fex* at the end of the experiment was similar (Fig. 3a). Also, the peak amounts of [^18^F]fluoride were comparable varying from plant to plant regardless of the presence of FEX (Fig. 2). The velocities of initial transport into the stem were similar in WT (2.2 $\pm$ 0.84 cm/min) and *fex* (2.4 $\pm$ 0.86 cm/min; Fig. 3b), but could be slowed in the WT (1.3 $\pm$ 0.28 cm/min) with the addition of 2.5 mM cold NaF (Fig. 3b).

The initial velocity of fluoride movement within *Arabidopsis* was slower than the rate of 36 cm/min originally calculated in soybean by McKay et al. (1988), the same as 2.2 cm/min reported for *Brassica oleracea* (Converse et al. 2015), and faster than tea (Niu et al. 2020). The speed was similar to that (1.9–1.1 cm/min) calculated for [^15^O]H_2_O in rice leaves (Kiyomiya et al. 2001). Although a portion of [^18^F]fluoride in the transpiration stream of the *fex* stem had an initial velocity equivalent to WT (2.4 cm/min), the vast majority of fluoride remained stationary, which affected the overall bulk movement.

The rate of initial transport up the stem was not affected by the FEX mutation, however the washout kinetics were very different. Fluoride clearance rates were calculated for WT and *fex* plants (Sup. Fig. S5 and S6). To directly compare WT and *fex* clearance rates, the value closest to 20 mm was chosen because it was close to the bolus entry site and it showed a clear pattern in the time activity curves, but it was far enough away to avoid background from the administration site. Comparison of the point directly above and below the 20 mm value showed the values were consistent, especially in the *fex* stem (Sup. Fig. S7a). The [^18^F]fluoride concentration decreased with time in WT plants, with clearance rates of 0.027 $\pm$ 0.012 min^−1^ at 20 mm up the stem, consistent with bulk flow. However, within the mutant, [^18^F]fluoride concentrations remained constant in lower portions of the plant and increased slowly at higher positions (Sup. Fig. S5 and S6). The clearance rate of 0.00095 $\pm$ 0.001 min^−1^ at the 20 mm position on the mutant stem was significantly slower than the WT rates (P < 0.0001) and consistent with a lack of continuous fluoride transport out of the tissue (Fig. 3c).

### [^18^F]Fluoride kinetic properties in WT and ***fex*** terminal tissues

**Fig. 4:**
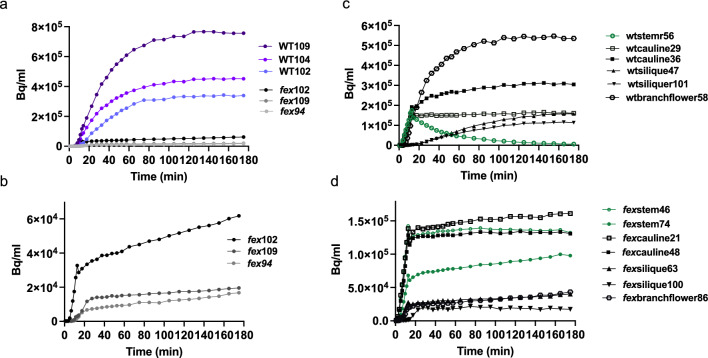
Time activity curve patterns of accumulation of [^18^F]fluoride in terminal tissues. **a** Accumulation in terminal flowers from 3 h PET scans. WT flowers are depicted in color, while mutant flower values, in black and grey, are too low to be visible on this graph. Numbers indicate mm from bottom of the field of view. **b** Expanded view of mutant terminal flower accumulation from **a**. **c** Time activity curves from a representative WT plant of siliques (triangle), branch flowers (hexagon), and cauline leaves (square) in comparison to stem sections (green circles). **d** TACs from a representative *fex* plant of siliques (triangle), branch flowers (hexagon), and cauline leaves (square) in comparison to stem sections (green circles)

Next, we analyzed [^18^F]fluoride movement from the stem into terminal tissues. [^18^F]Fluoride was efficiently cleared even in the uppermost WT stem sections (Fig. 2a). At the apex is the terminal flower cluster. We have previously shown that over the lifespan of the plant, fluoride accumulates to high levels in floral buds in *fex*, but not WT, plants causing sterility (Tausta et al. 2021). To evaluate [^18^F]fluoride dynamics in terminal tissues, three hour scans were used with the terminal flower placed well within the frame of view. From the last frame of the PET image, it was evident that [^18^F]fluoride accumulated in all the terminal WT tissues including the cauline leaves, branch and terminal flowers, and siliques (Fig. 1b, Sup. Movie1). WT terminal flowers accumulated [^18^F]fluoride which reached a maximal concentration after approximately 1.5 h (Fig. 4a). Cauline leaves and siliques had different patterns of [^18^F]fluoride accumulation in the WT plant (Fig. 4c). Accumulation in WT cauline leaf tips was evident when leaves were favorably positioned during the PET scan consistent with known tip accumulation of fluoride. WT cauline leaves quickly (~ 20 min) accrued [^18^F]fluoride which was not cleared. WT siliques gained [^18^F]fluoride more slowly than leaves and continued to accumulate for at least 2 h. Although there were slight differences in the pattern of accumulation of [^18^F]fluoride in the WT, there appeared to be no selective accumulation in a single tissue type. Unlike [^18^F]fluoride movement in the WT stem sections, there appeared to be no clearance of the radioactivity from terminal tissues during this time period under these experimental conditions.

In *fex* terminal flowers, [^18^F]fluoride concentrations were an order of magnitude lower and gradually increased throughout the 3 h scan (Fig. 4a and b). Unlike the WT plant, only a small fraction of the [^18^F]fluoride was usually found in the *fex* terminal flowers after 3 h. Flowers at the ends of branches showed a pattern of [^18^F]fluoride dynamics similar to the terminal flowers (Fig. 4c and d). The same pattern of radioactivity accumulation as the stem sections was seen for the mutant cauline leaves and siliques. This included the trend where more radioactivity was seen lower on the plant regardless of the type of tissue (Fig. 4d).

Fluoride movement swiftly up the floral stem and accumulation in the flowers of WT plants was unexpected given our previous result of low fluoride exposure over an extended time period (weeks). Due to the short half-life of [^18^F]fluoride, scans of a significantly longer duration were not feasible. Based on the observation that WT flowers accumulated fluoride on the time scale of hours, but did not on the time scale of weeks, we hypothesized that [^18^F]fluoride may be leaving the WT plant. To test this hypothesis, we analyzed the cold buffer bath used to wash out the initial bolus, but no radioactivity was detectable, suggesting fluoride likely does not exit through the stem in three hours. Further, when a lateral branch was cut off, PET images revealed that radioactivity had leaked from the wound, suggesting that the majority of radioactivity moved towards the plant apex in the transpiration stream rather than towards the base. No radioactivity was detected on filters placed under the terminal flower during the scan. This suggests that fluoride leaving the plant in the WT is likely on a time scale longer than hours, but shorter than weeks and/or under growth conditions different than those analyzed in this study.

### Cold fluoride can inhibit [^18^F]fluoride movement in WT plants

**Fig. 5:**
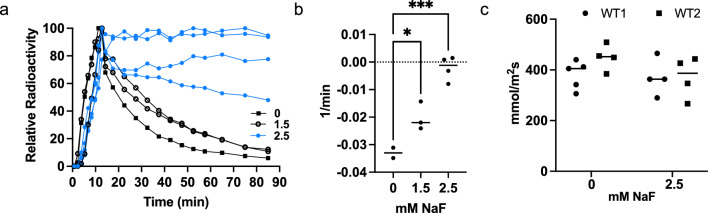
NaF treatment inhibited [^18^F]fluoride movement in WT plants. **a** Normalized TAC of treatments of NaF 24 h before and during the [^18^F]fluoride pulse given to the stems of WT plants. Open circles represent treatment with 1.5 mM NaF and blue circles represent 2.5 mM NaF. **b** Results of the clearance rate calculation for the data in a. One way ANOVA (Dunnett’s) where * is P = 0.0310 and *** is P = 0.0004. **c** Transpiration rate remained unchanged upon treatment of 2.5 mM NaF for 24 h. Two-way ANOVA all values not significant (ns)

The kinetics of [^18^F]fluoride suggested that FEX may mediate a fluoride transport mechanism throughout the entire plant. To test if this mechanism was saturable, WT plants grown hydroponically were treated with a large bolus of unlabeled fluoride at the same time as the [^18^F]fluoride. Addition of 2.5 mM cold fluoride to the 1.5 nM [^18^F]fluoride tracer resulted in the accumulation of [^18^F]fluoride in the stem that was not efficiently cleared (Fig. 5a). The WT pattern with high fluoride was similar to that observed for *fex* plants without added cold fluoride. Quantitative analysis of the WT clearance rates found slower [^18^F]fluoride clearance at 1 mM NaF compared to when no cold fluoride was added and negligible clearance upon 2.5 mM NaF treatment similar to the near-zero rates determined for the *fex* curves generated without cold fluoride (P = 0.0006; Fig. 5b). Concentrations of 2.5 mM NaF have been shown to be sufficient to cause phenotypic changes such as stunted growth in WT *Arabidopsis* seedlings (Tausta et al. 2021). The similarity of the WT activity curve at high fluoride concentration compared to mutant curve at trace concentration suggests that the FEX transporter function is saturable.

High concentrations of fluoride are known to cause wilting and inhibition of transpiration (Kamaluddin and Zwiazek 2003; Elloumi et al. 2017). The concentrations or time of exposure used in these experiments were comparable to those found to be inhibitive in other plants (*Zea mays*: 10 mM KF 5 min, *Lycopersicon esculentum*/tomato: 100 mM KF 5 min; Yamauchi et al. 1983; *Populus tremuloides Michx./*aspen: 0.3 mM NaF 3wk; Kamaluddin and Zwiazek 2003; *Salix viminalis*/willow: EC_50_ 2.5 mM 96 h; Clausen et al. 2015). The transpiration rate of WT plants treated with 2.5 mM NaF did not change after 24 h (Fig. 5c). There was no indication of wilting during this time period. Much higher concentrations did cause visible wilting and are not reported here.

We also attempted to alter [^18^F]fluoride kinetics in WT plants with the anion channel blocker NPPB at 50 and 100 µM, which had been shown to inhibit fluoride uptake in tea (Zhang et al. 2016). No large change in the pattern of [^18^F]fluoride movement in the WT plant was detected (Sup. Fig. S5b).

## Discussion

**Fig. 6:**
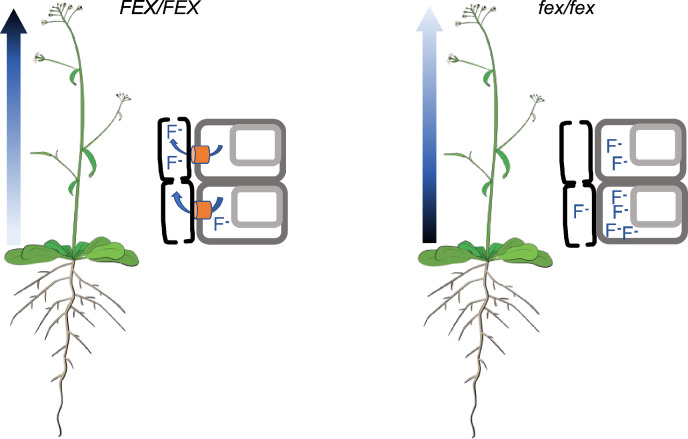
A depiction of fluoride movement in WT and *fex* plants. In the 3 h of the PET experiments, fluoride rapidly flowed up the WT floral stem, but not the *fex* stem resulting in high concentrations in WT terminal tissues and in basal tissues in the *fex* plants as represented by the color gradient arrow. The best explanation for this pattern of fluoride movement is that FEX in the cell membrane (orange cylinders) exports fluoride quickly into the transpiration stream. If FEX is not present, fluoride is not effluxed, increasing the steady state concentration of fluoride in the cytoplasm to the detriment of the cell

Dynamic observation of [^18^F]fluoride via PET revealed that FEX is involved in the movement and accumulation of fluoride throughout the plant. FEX appears to be responsible for directed fluoride movement in the plant and that fluoride movement with the transpiration stream is the basis for WT fluoride tolerance (Fig. 6). In WT plants, [^18^F]Fluoride accumulated in all terminal tissues. From the patterns of [^18^F]fluoride movement, the roots do not appear to be the primary locus of FEX action nor does it seem that FEX directed [^18^F]fluoride to a specific tissue or immediately sequester [^18^F]fluoride into the vacuoles. The data are most consistent with FEX acting in each individual cell to provide overall fluoride tolerance to the whole plant.

The PET experiments allowed the evaluation of several models for the function of FEX in fluoride tolerance as described: vacuolar localization, avoidance, selective tissue targeting, and each cell for itself.

It is well-established that many toxins are shunted to the vacuoles, which function as depositories for harmful substances, including toxic ions. The PET data indicate that vacuole detoxification is not a primary mechanism of fluoride resistance in *Arabidopsis*, at least not in the first three hours of exposure. This model predicts that [^18^F]fluoride would not move with the transpiration stream in WT plants, but would be shunted to the vacuoles and appear to be stationary. The anion Cl^−^ has been shown to be shunted into barley vacuoles on the order of 12 min (Martinoa et al. 1986). We observe the opposite for [^18^F]fluoride in WT *Arabidopsis* floral stems. It was not expected that FEX would function in the vacuoles because of the rescue of the membrane FEX in *S. cerevisiae* (Li et al. 2013) and because AtFEX has been identified as a cell membrane protein (SUBA4; Hooper et al. 2017). Yet, a role for FEX in the vacuolar membrane cannot be fully excluded. It might play a role at higher fluoride concentrations, later times, under different environmental conditions, or within specific parts of the plant. It may even vary between different plant species. *Arabidopsis* is an herbaceous annual with a short life cycle. Plants with different growth habits might react differently like tea, a woody evergreen, which does show slow movement of [^18^F]fluoride from the roots into the stem (Niu et al. 2020). Fluoride has been detected in tea leaf vacuoles (Gao et al. 2014). An alternative interpretation of our cold fluoride experiment is that the higher concentration of fluoride activates a mechanism where fluoride is shunted to the vacuoles and appears to not progress up the stem. Tea has an additional FEX homolog that is expressed when fluoride levels increase and could be responsible for moving fluoride into vacuoles (Song et al. 2020). Another indication that tea might have additional fluoride detoxification methods is the finding that anion inhibitors like NPPB reduce the intake of fluoride (Zhang et al. 2016). We did not observe a similar effect in *Arabidopsis,* which is less fluoride-tolerant than tea. Further research into FEX expression and cell location in fluoride susceptible and fluoride tolerant plants will be needed when contemplating the engineering of susceptible plants to tolerate environmental fluoride.

The other three models for fluoride tolerance proposed here assume that FEX functions within the plasma membrane, a location for which there is clear evidence. FEX homologs from several different plants all rescued a yeast FEX knockout mutant, where FEX is known to function within the cell membrane (Berbasova et al. 2017; Zhu et al. 2019; Tausta et al. 2021). Also, GFP-labeled tea FEX appears in the root cell membranes of transformed *Arabidopsis* (Zhu et al. 2019).

In the ‘avoidance’ model, the roots are expected to provide the primary location of fluoride efflux by preventing entry of fluoride into the transpiration stream. Plants often have multiple transporters to maintain optimal levels of a substance within cells. For instance, there are more than 10 known transporters of chloride and some in the nitrate excretion transporter (NXT) family are responsible for chloride efflux specifically from root cells (Li et al. 2017). Roots of some varieties of citrus or grape are so efficient at keeping chloride out of the shoot that they are used as root stock for more susceptible varieties (Brumós et al. 2010; Tregeagle et al. 2010; Henderson et al. 2014). It appears that only the single *Arabidopsis* FEX gene is responsible for fluoride tolerance. Two results argue against the avoidance strategy. First, both WT and *fex* plants accumulated similar amounts of [^18^F]fluoride when the bolus was applied through the intact roots. Second, the pattern of fluoride distribution was not altered for either the WT or *fex* plants when the fluoride bolus was applied through the roots or directly into the stem. Although we cannot conclude there is no role for the roots in fluoride efflux, it does not appear to be the primary mechanism of protection from fluoride toxicity in *Arabidopsis*.

The third model proposes that FEX directs fluoride to a less vulnerable or dispensable tissue within the plant or selective tissue targeting. An example of this is the accumulation of zinc in the leaf trichomes of *Arabidopsis thaliana* and the hyperaccumulation of zinc in *Arabidopsis halleri* (Zhao et al. 2000; Ricachenevsky et al. 2021). Trichomes on leaves are a convenient noncrucial tissue near the end of the transpiration stream with the added benefit of being a deterrent to herbivory. During the period of the PET experiments, there was no evidence of [^18^F]fluoride being directed to a specific tissue. [^18^F]Fluoride accumulated into all terminal tissues, including vulnerable reproductive tissues. Thus, the PET data are inconsistent with the selective tissue targeting model, at least during the first hours of exposure.

The PET results are most consistent with a model in which each cell appears to be acting for itself to keep fluoride moving through the transpiration stream (Fig. 6). This is similar to the action of FEX within single-celled organisms where efflux out of each individual cell relieves toxicity. It is also consistent with previous experiments which determined that overexpression of *FEX* led to increased tolerance to environmental fluoride and that *FEX* is expressed in most tissues (Zhu et al. 2019; Song et al. 2020; Tausta et al. 2021). *FEX* expression is prominent in young tissues, as demonstrated by both RT-PCR and promoter GUS experiments, and shows some increase upon fluoride exposure (Zhu et al. 2019; Song et al. 2020; Tausta et al. 2021). In the FEX mutant, the [^18^F]fluoride entered the plant vasculature at the same rate as WT, but did not move with the bulk water up the stem of the plant. This same pattern was observed for WT plants exposed to high concentrations of cold fluoride, consistent with a model in which the action of FEX is saturable. In the absence of FEX or upon FEX saturation, fluoride accumulates in cells. The pattern of fluoride immobility suggests that the mutant cells are unable to efflux fluoride. Reports using radioactive water showed that much of the water traveling in the xylem actually leaks out horizontally and then re-enters the transpiration stream to continue up the plant (Ohya et al. 2008; Metzner et al. 2010). Fluoride would be expected to stay solvated and travel with the water into surrounding tissues. With a functioning FEX, fluoride localized into xylem-adjacent cells would be effluxed back into nearby vessels, allowing it to continue movement through the plant with the bulk water (Ohya et al. 2008; Metzner et al. 2010). This explains the faster apical movement of [^18^F]fluoride within the WT plant.

At later time points, [^18^F]fluoride did slowly move up the mutant stem and into the terminal flower. When grown for weeks in substrates containing very low (~ 1 µM) concentrations of fluoride, mutant plants did accumulate fluoride in the flowers (Tausta et al 2021). While the physical half-life of ^18^F prevents scanning for multiple days, future work could examine the accumulation of [^18^F]fluoride with longer time periods (e.g., up to 12 h). Fluoride immobility in *fex* plants would increase the steady-state fluoride concentration within mutant cells resulting in more cellular damage (Zhu et al. 2019; Tausta et al. 2021). Early experiments have suggested that plants vary in their susceptibility to fluoride toxicity due to differences in speed of movement within the plant with the slowest (gladiolus) being the most susceptible and the fastest (cotton) being the least (Jacobson et al. 1966).

The accumulation of [^18^F]fluoride into terminal flowers in the WT plant was an unexpected result given our previous work on the *fex* mutant. We found that the mutant, and not WT, plants accumulated fluoride in the flowers when grown for long periods (weeks) in low-fluoride-containing substrates. While the ‘each cell for itself’ model establishes how individual cells reduce fluoride damage, there must also be a mechanism to rid the plant of accumulated fluoride once it reaches the terminal tissues.

There are two ways in which water exits the plant; as vapor from the stomata and as liquid from the hydathodes. These are the most likely mechanisms for some fluoride release from the plant. It was previously reported that fluoride accumulation in the tips of cotton leaves was also accompanied by the appearance of more fluoride on the external leaf surface suggesting an ability to release fluoride from internal tissues in cotton (Jacobson et al. 1966). Water vapor released from stomata can contain volatiles like methanol (Hüve et al. 2007). HF, in particular, is highly soluble in water and could be emitted through the stomata. Ions and other substances have also been detected in the guttation exudate and it is considered a method of reducing concentrations from within the plant (Singh and Singh 2013). Our PET experiments establish that there is efficient movement of [^18^F]fluoride to the *Arabidopsis* WT terminal tissues via the transpiration stream. Although this is consistent with the fact that leaf tips are where fluoride damage is seen in most plants, it could also suggest that, like cotton, fluoride can be released into the atmosphere via hydathodes and/or stomata. Fluoride ions were found in the guttation fluid of maize. However, it was determined that the amount detected there was not sufficient to account for the total loss of fluoride in leaves (Takmaz-Niscancioglu and Davison 1988). The presence of fluoride in the guttation fluid suggests it is possible for elimination to occur via this route (Weinstein 1977).

The comparison of [^18^F]fluoride movement kinetics in FEX mutant and WT plants has revealed the inability of the mutant to flush fluoride from tissues during transpiration on the time scale of hours, which clarifies how plants deal with this environmental toxin. The PET results are most consistent with a model of FEX action by each cell for fluoride tolerance. These results underscore the importance of this one transporter in protecting the entire plant from fluoride toxicity.

### Supplementary Information

Below is the link to the electronic supplementary material.

Supplemental Table 1 Listed are all the experiments performed for this publication. Root means the bolus was applied to the roots and the roots remained intact for the whole trial. Root to stem means the bolus was applied to the roots and then after the 10min the roots were cut off and the stem put in cold buffer. Stem to stem means the bolus was added to the cut stem and after the 10min the stem was recut and put in cold buffer. Supplemental Figure 1 Time Activity Curves (TACs) resulting from a 10min [^18^F]fluoride bolus in Bq/ml from sections of the stem denoted by the mm at the midpoint of the ROI measured from the bottom of the field of view. a An example of 3 WT cut stem experiments from which curve fits were calculated for determination of the clearance rates. b The TAC from root application of bolus followed by washing and leaving the roots intact in both the WT and fex plants. The patterns of fluoride movement in each genotype at 15mm are different. c The TAC of a trial where [^18^F]fluoride was applied to the roots followed by cutting and inserting the stem in cold buffer. The ROI closest to 20 mm was chosen to visualize [^18^F]fluoride flow. The patterns are sharper than in b. Supplemental Figure 2 Depiction of floral stems and PET scans with ROI placement that resulted in the TACs found in Figure 2. On the left of each panel is a picture of the floral stem on the acrylic plate before gauze was wrapped around the plant and plate prior to the PET scan. On the right is the last PET frame on which ROIs were drawn. a The WT plant which was used to generate Fig. 2a. b The fex plant used to generate the TAC in Fig. 2c. Supplemental Figure 3 Confocal images of pADH1:YFP-AtFEX rescued fex plants. Plant tissues pictured are labeled on the left and detection is across the top. A seedling (7d) showing strong expression in young vegetative tissue (size bar 100 µM). In a mature leaf, epidermal cells are lightly YFP positive, while the mesophyll cells exhibit strong signal (size bar 50 µM). Roots have light staining (size bar 50 µM). In the cross section of the floral stem, the green tissue has a strong signal and other cell types display low or no signal (size bar 100 µM). The flower bud sepals show a strong signal, but the petals do not (size bar 100 µM). Supplemental Figure 4 Transpiration rates of WT and fex plants grown in soil substrate are similar. Plants were grown to the age at which PET experiments were performed and the mutants were beginning to show fluoride toxicity and then leaves on different plants were monitored for transpiration. Supplemental Figure 5 Clearance rates for all ROIs in WT stems. The clearance rates (min-1) for each ROI beginning with the lowest and proceeding up the floral stem are graphed. Note that all clearance rates are below 0. Supplemental Figure 6 Clearance rates for all ROIs in fex floral stems. The clearance rates (min-1) for each ROI beginning with the lowest and proceeding up the stem are graphed. At the stem bottom the rate is below zero, but at ROIs further up the stem the clearance rates increase and become positive. Supplemental Figure S7 a Clearance rate around 20mm ROI. The clearance rates closest to ROI 20 mm plus the ROIs above and below are graphed. Values from WT plants are black circles and values from fex plants are open circles. b Normalized TACs of NPPB treatment for 24 h before and during the [^18^F]fluoride pulse which was given to the roots and then cut off and the stem inserted into cold buffer. Supplementary file1 (PDF 854 KB)

Supplemental Movie1 Dynamic PET data from [^18^F]fluoride bolus applied to cut stem followed by wash with fluoride-free buffer. This is the same trial as shown in Fig. 1b with fex on the left and WT on the right. The movie is looped. Supplementary file2 (MP4 6969 KB)

## Data Availability

Enquiries about data availability should be directed to the authors.
