# Supplementary material for: Fluoride transport in Arabidopsis thaliana plants is impaired in Fluoride EXporter (FEX) mutants
Source: Plant Mol Biol. 2024 Feb 11;114(1):17. doi: 10.1007/s11103-023-01413-w (PMC10859346; doi:10.1007/s11103-023-01413-w)

Supplemental

Supplemental Movie1

Supplemental Table S1

Genotype	Bolus site	Experimental conditions	trials
FEX/FEX	root	standard	4
FEX/FEX	root	continuous/no wash out	2
FEX/FEX	root	plus 500µM cold NaF	1
FEX/FEX	root to stem	standard	3
FEX/FEX	stem to stem	standard	12
FEX/FEX	stem to stem	plus 350mM NaF	2
FEX/FEX	stem to stem	plus 1mM NaF	1
FEX/FEX	stem to stem	plus 1.5mM NaF	2
FEX/FEX	stem to stem	plus 2.5mM NaF	4
FEX/FEX	root	plus 50µm NPPB	5
FEX/FEX	root	plus 100µm NPPB	2
fex/fex	root	standard	4
fex/fex	root	plus 500µM cold NaF	1
fex/fex	root	plus 50µM NPPB	3
fex/fex	root to stem	standard	3
fex/fex	stem to stem	standard	10

Supplemental Figure S1

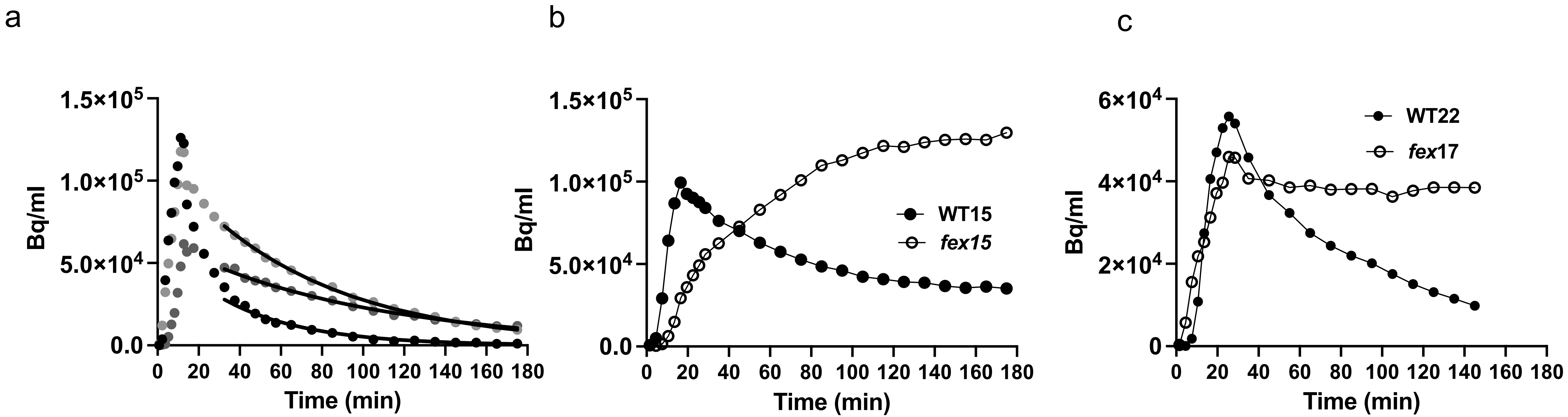

Supplemental Figure S2

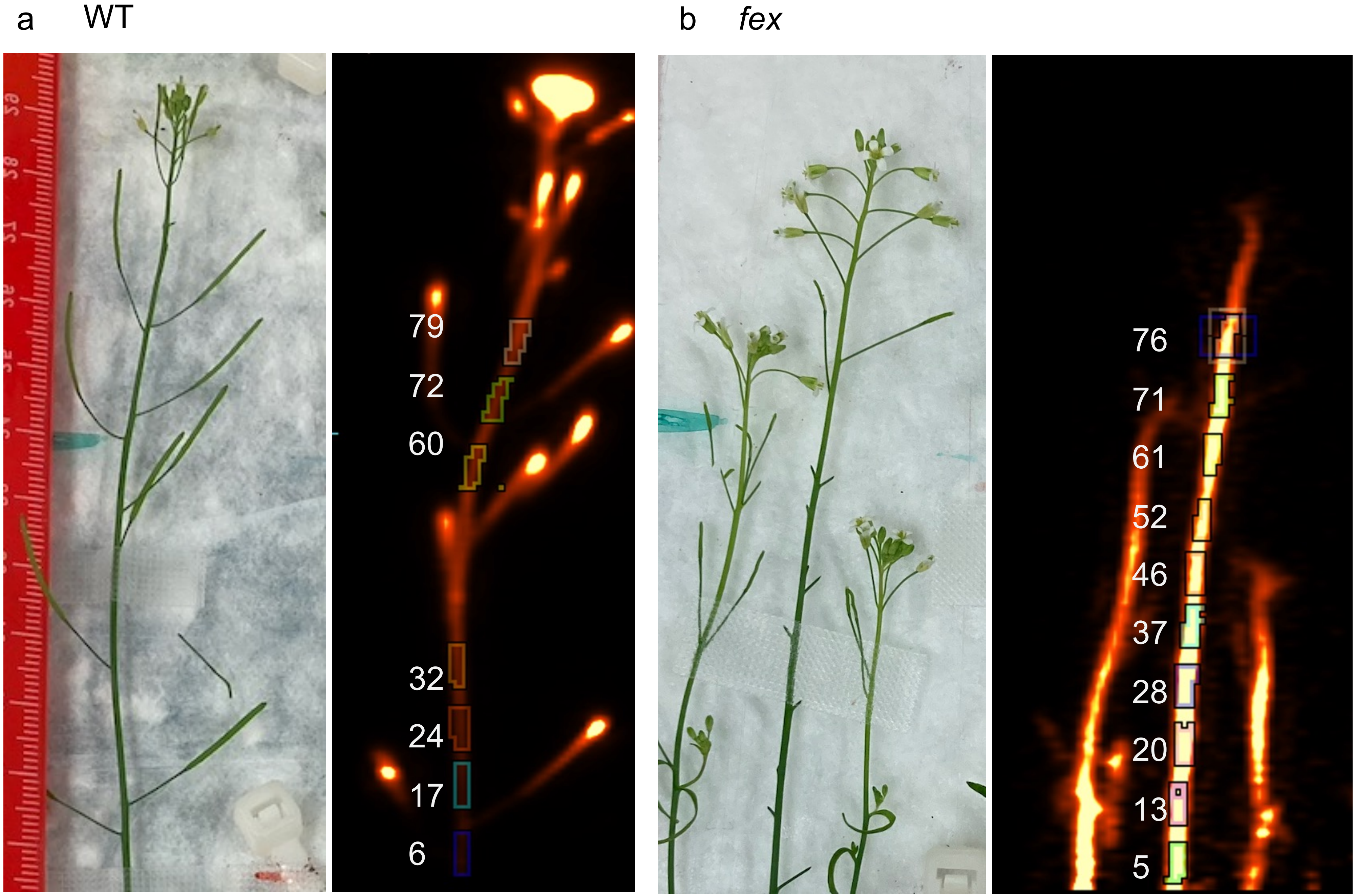

Supplemental Figure S3

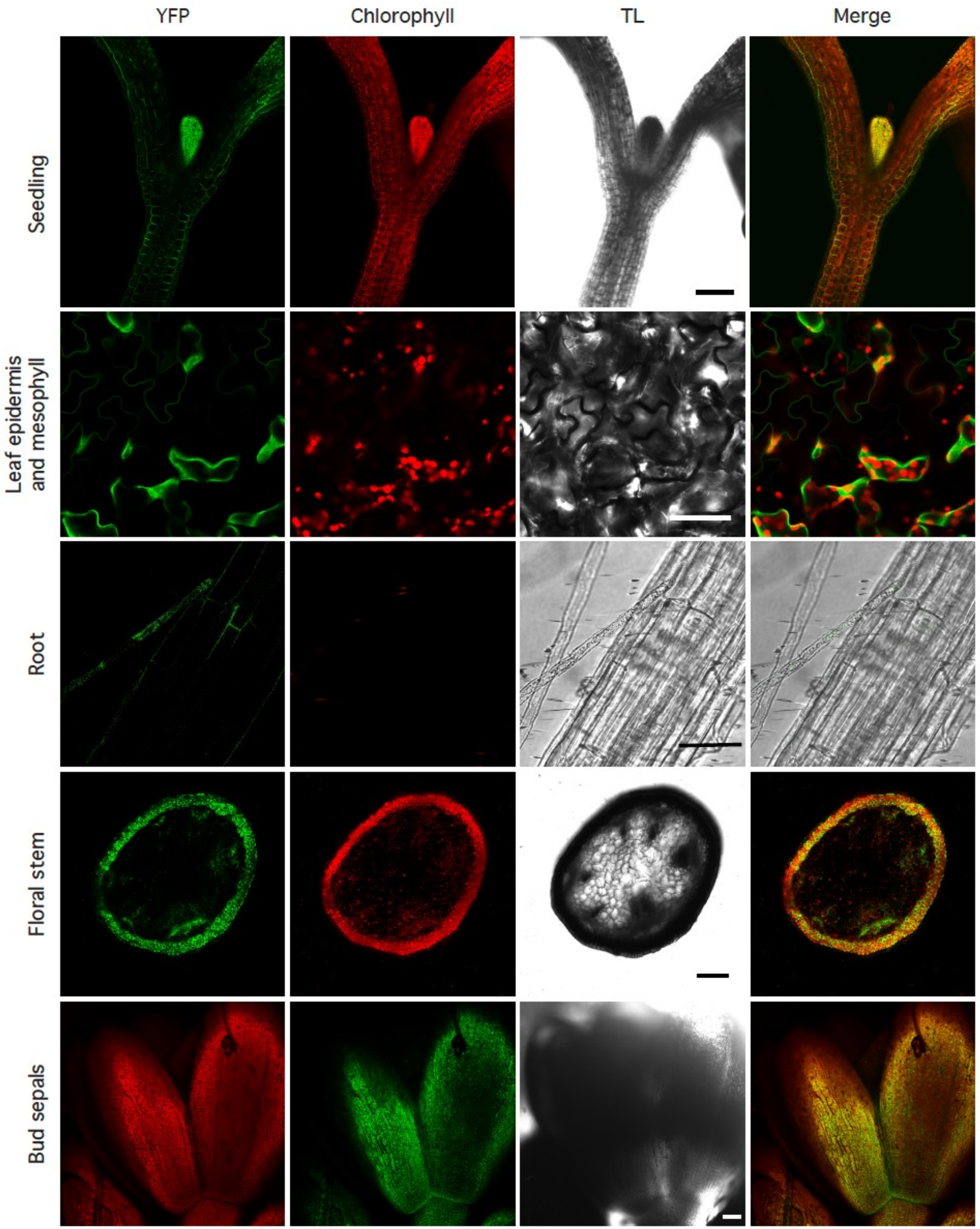

Supplemental Figure S4

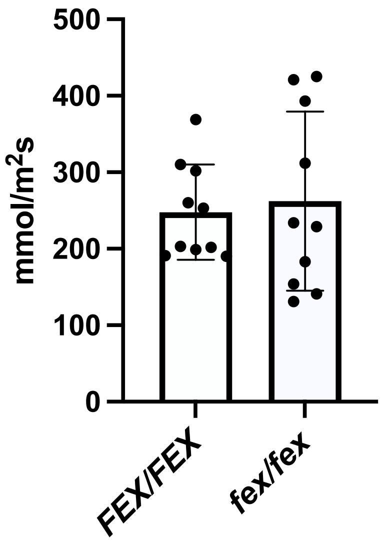

Supplemental Figure S5

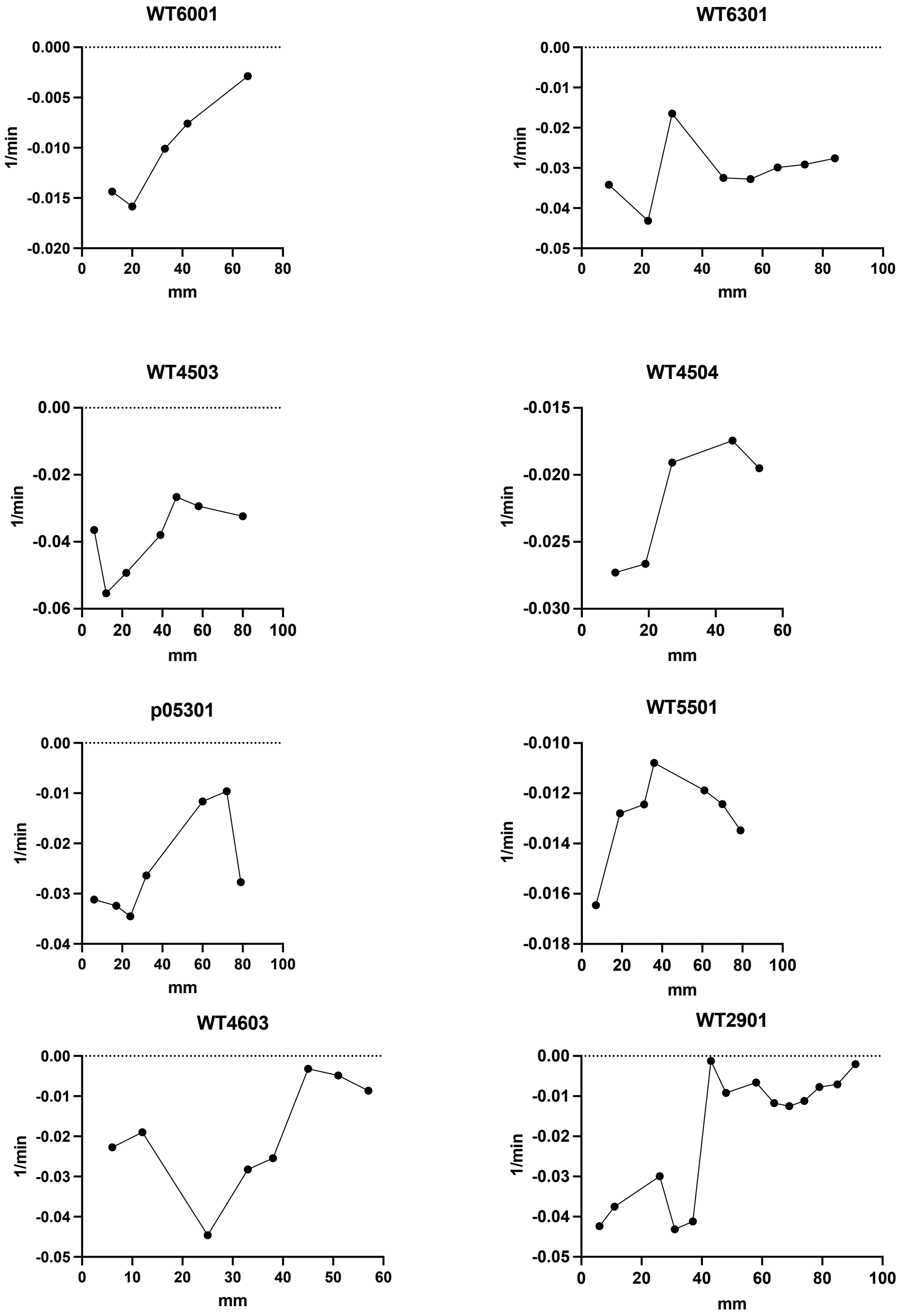

Supplemental Figure S6

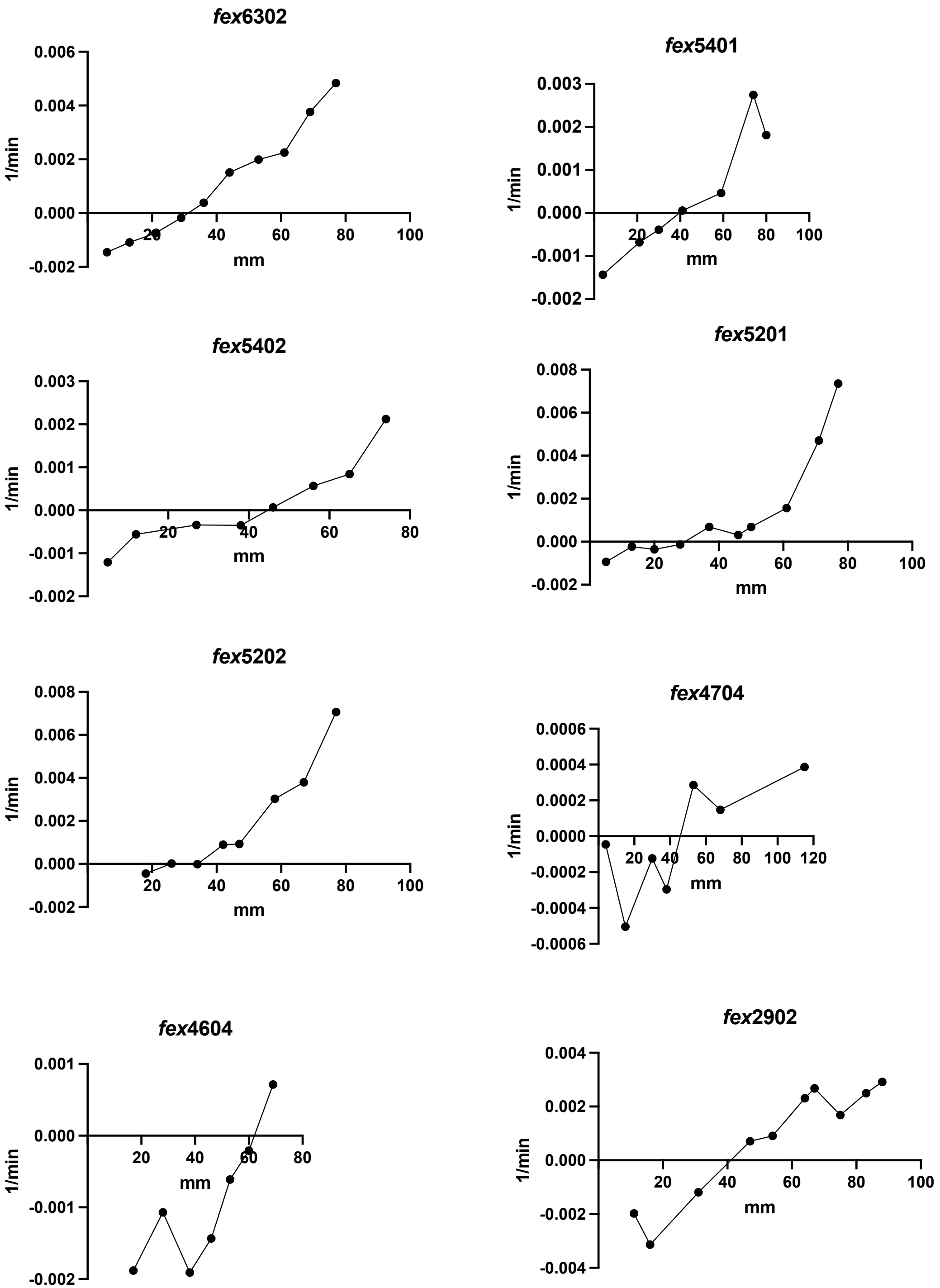

Supplemental Figure S7

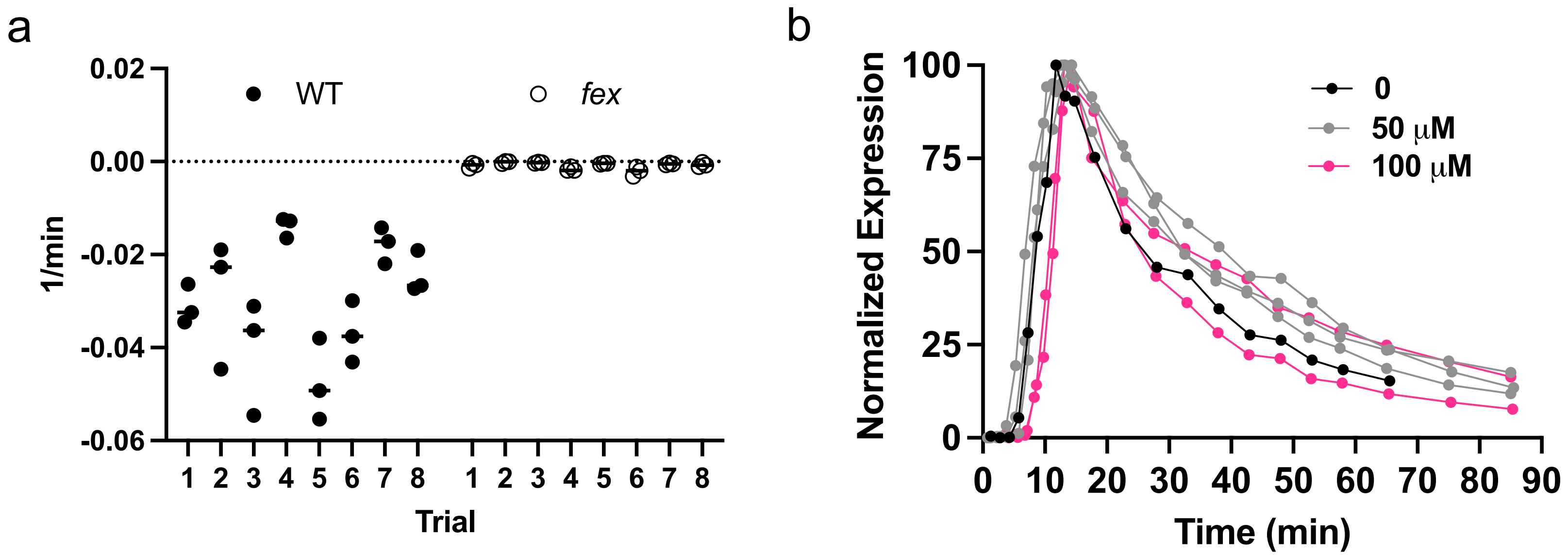

Supplement: Supplementary file 1 — Supplemental Table 1 Listed are all the experiments performed for this publication. Root means the bolus was applied to the roots and the roots remained intact for the whole trial. Root to stem means the bolus was applied to the roots and then after the 10min the roots were cut off and the stem put in cold buffer. Stem to stem means the bolus was added to the cut stem and after the 10min the stem was recut and put in cold buffer. Supplemental Figure 1 Time Activity Curves (TACs) resulting from a 10min [18F]fluoride bolus in Bq/ml from sections of the stem denoted by the mm at the midpoint of the ROI measured from the bottom of the field of view. a An example of 3 WT cut stem experiments from which curve fits were calculated for determination of the clearance rates. b The TAC from root application of bolus followed by washing and leaving the roots intact in both the WT and fex plants. The patterns of fluoride movement in each genotype at 15mm are different. c The TAC of a trial where [18F]fluoride was applied to the roots followed by cutting and inserting the stem in cold buffer. The ROI closest to 20 mm was chosen to visualize [18F]fluoride flow. The patterns are sharper than in b. Supplemental Figure 2 Depiction of floral stems and PET scans with ROI placement that resulted in the TACs found in Figure 2. On the left of each panel is a picture of the floral stem on the acrylic plate before gauze was wrapped around the plant and plate prior to the PET scan. On the right is the last PET frame on which ROIs were drawn. a The WT plant which was used to generate Fig. 2a. b The fex plant used to generate the TAC in Fig. 2c. Supplemental Figure 3 Confocal images of pADH1:YFP-AtFEX rescued fex plants. Plant tissues pictured are labeled on the left and detection is across the top. A seedling (7d) showing strong expression in young vegetative tissue (size bar 100 µM). In a mature leaf, epidermal cells are lightly YFP positive, while the mesophyll cells exhibit strong signal ( [file 11103_2023_1413_MOESM1_ESM.pdf]
